# *In vitro* model to study the effects of matrix stiffening on Ca^2+^ handling and myofilament function in isolated adult rat cardiomyocytes

**DOI:** 10.1113/JP274460

**Published:** 2017-06-21

**Authors:** Elza D. van Deel, Aref Najafi, Dulce Fontoura, Erik Valent, Max Goebel, Kim Kardux, Inês Falcão‐Pires, Jolanda van der Velden

**Affiliations:** ^1^ Department of Physiology, Amsterdam Cardiovascular Sciences VU University Medical Center Amsterdam the Netherlands; ^2^ Department of Surgery and Physiology, Faculty of Medicine Universidade do Porto Portugal; ^3^ Netherlands Heart Institute Utrecht the Netherlands

**Keywords:** cardiomyocyte function, cell culture, experimental model, extracellular matrix

## Abstract

**Key points:**

- This paper describes a novel model that allows exploration of matrix‐induced cardiomyocyte adaptations independent of the passive effect of matrix rigidity on cardiomyocyte function.
- Detachment of adult cardiomyocytes from the matrix enables the study of matrix effects on cell shortening, Ca^2+^ handling and myofilament function.
- Cell shortening and Ca^2+^ handling are altered in cardiomyocytes cultured for 24 h on a stiff matrix.
- Matrix stiffness‐impaired cardiomyocyte contractility is reversed upon normalization of extracellular stiffness.
- Matrix stiffness‐induced reduction in unloaded shortening is more pronounced in cardiomyocytes isolated from obese ZSF1 rats with heart failure with preserved ejection fraction compared to lean ZSF1 rats.

**Abstract:**

Extracellular matrix (ECM) stiffening is a key element of cardiac disease. Increased rigidity of the ECM passively inhibits cardiac contraction, but if and how matrix stiffening also actively alters cardiomyocyte contractility is incompletely understood. *In vitro* models designed to study cardiomyocyte–matrix interaction lack the possibility to separate passive inhibition by a stiff matrix from active matrix‐induced alterations of cardiomyocyte properties. Here we introduce a novel experimental model that allows exploration of cardiomyocyte functional alterations in response to matrix stiffening. Adult rat cardiomyocytes were cultured for 24 h on matrices of tuneable stiffness representing the healthy and the diseased heart and detached from their matrix before functional measurements. We demonstrate that matrix stiffening, independent of passive inhibition, reduces cell shortening and Ca^2+^ handling but does not alter myofilament‐generated force. Additionally, detachment of adult cultured cardiomyocytes allowed the transfer of cells from one matrix to another. This revealed that stiffness‐induced cardiomyocyte changes are reversed when matrix stiffness is normalized. These matrix stiffness‐induced changes in cardiomyocyte function could not be explained by adaptation in the microtubules. Additionally, cardiomyocytes isolated from stiff hearts of the obese ZSF1 rat model of heart failure with preserved ejection fraction show more pronounced reduction in unloaded shortening in response to matrix stiffening. Taken together, we introduce a method that allows evaluation of the influence of ECM properties on cardiomyocyte function separate from the passive inhibitory component of a stiff matrix. As such, it adds an important and physiologically relevant tool to investigate the functional consequences of cardiomyocyte–matrix interactions.

## Key points

- This paper describes a novel model that allows exploration of matrix‐induced cardiomyocyte adaptations independent of the passive effect of matrix rigidity on cardiomyocyte function.
- Detachment of adult cardiomyocytes from the matrix enables the study of matrix effects on cell shortening, Ca^2+^ handling and myofilament function.
- Cell shortening and Ca^2+^ handling are altered in cardiomyocytes cultured for 24 h on a stiff matrix.
- Matrix stiffness‐impaired cardiomyocyte contractility is reversed upon normalization of extracellular stiffness.
- Matrix stiffness‐induced reduction in unloaded shortening is more pronounced in cardiomyocytes isolated from obese ZSF1 rats with heart failure with preserved ejection fraction compared to lean ZSF1 rats.

**Abbreviations**
APTES: 3‐aminopropyltrimethoxysilane
Bis: *N*,*N*′‐methylene‐bis‐acrylamide
ECM: extracellular matrix
*F*_max_: myofilament maximal force
*F*_pas_: myofilament passive force
K_tr_: maximal rate of force redevelopment
EC_50_: myofilament Ca^2+^ sensitivity
HFpEF: heart failure with preserved ejection fraction
Sulpho SANPAH: sulfosuccinimidyl 6‐(4′‐azido‐2′‐nitrophenylamino)hexanoate

## Introduction

The extracellular matrix (ECM) is one of the key players in cardiac remodelling and dysfunction in the diseased heart (Weber, 1997; Mewton *et al*. 2011; Wong *et al*. 2012; Schelbert *et al*. 2014). Stiffening of the ECM impairs cardiac function by passive inhibition of cardiac contractility. In addition to that, an increasing number of studies suggest that mechanical properties of the ECM also actively influence cardiomyocyte pathology (Engler *et al*. 2008; Forte *et al*. 2012; Galie *et al*. 2013; Hersch *et al*. 2013). As such the ECM is a promising therapeutic target for improving cardiac function in the diseased heart (Piek *et al*. 2016). Mechanical properties of the ECM are directly sensed by the sarcomeres, via the costamere complex (Galie *et al*. 2013), but also enter the cells via the microtubule cytoskeleton (Robison *et al*. 2016) in a process known as mechanotransduction. However, the mechanisms and impact of cardiomyocyte–matrix interactions in cardiac pathology are still poorly understood. In the whole heart, multiple factors (e.g. matrix stiffness, reactive oxygen species, matricellular proteins, transforming growth factor β, chemokines and cytokines) (Kong *et al*. 2014) underlie cardiomyocyte dysfunction. Investigation of the impact of matrix stiffening on cardiomyocyte function, in the absence of other ECM‐related factors, requires *in vitro* models in which only the stiffness of the extracellular environment is altered. Using matrices of tuneable stiffness, mimicking the healthy and the diseased heart, these models revealed stiffness‐related effects in stem cell differentiation (Engler *et al*. 2006; Even‐Ram *et al*. 2006), cell shape (McCain *et al*. 2014), beating frequency (Engler *et al*. 2008) and contraction (Galie *et al*. 2013; Jian *et al*. 2014). However, in the latter *in vitro* models cardiomyocytes were still attached to their matrix during cardiomyocyte measurements, and thus it was not possible to distinguish between the passive inhibitory effect of a stiff matrix and stiffness‐induced cardiomyocyte changes. To define ECM‐induced changes in cardiomyocyte function, an *in vitro* model is needed in which the cells are initially exposed to a specific matrix, but no longer attached to the matrix at the time of functional cardiomyocyte measurements. Yet, commonly used methods of detaching adherent cells from their matrices are ineffective and even lethal to adult cardiomyocytes.

Here we demonstrate a novel, reproducible and easy to use model of isolated adult cardiomyocytes that are cultured on polyacrylamide gels of defined tuneable stiffness (mimicking the healthy and the diseased heart) and detached from their matrices before functional measurements. This newly developed strategy of detaching isolated adult cardiomyocytes after exposure to different matrices opens up the possibility for functional measurements that could not be performed previously. The method makes it possible to study the effects of a matrix on the function of single intact (cell shortening, Ca^2+^ transients) and membrane‐permeabilized (myofilament properties) cardiomyocytes. Moreover, adult cardiomyocytes can be transferred from one matrix to another to evaluate the effect of altered matrix properties on cardiomyocyte function, and enables assessment of the effect of a stiff matrix on cardiomyocytes from disease models.

This novel approach of culturing adult cardiomyocytes on matrices of tuneable stiffness and subsequent detachment of the cells improves and expands the possibilities for studying cardiomyocyte–ECM interaction and its role in cardiac disease.

## Methods

The animal experiments were performed in accordance with the guidelines from Directive 2010/63/EU of the European Parliament on the protection of animals used for scientific purposes and approved by the ethics committees of the Faculty of Medicine of Porto, Portugal and VU medical centre in Amsterdam, the Netherlands. All procedures were in accordance with institutional guidelines. In total 14 wild‐type male Wistar rats (200 g), 2 male lean, non‐diabetic ZSF1 rats (28 weeks) and 5 male obese, diabetic ZSF1 rats (28 weeks) were used in the described experiments.

### Animal model of heart failure with preserved ejection fraction

To assess the impact of cardiac stiffness on diseased cardiomyocytes, we used 28‐week‐old ZSF1 obese rats (*n* = 5), which are diabetic, hypertensive and develop heart failure with preserved ejection fraction (HFpEF) (Hamdani *et al*. 2013), and compared them to ZSF1 lean rats (*n* = 2), which are only hypertensive and serve as controls. The ZSF1 animals were kept individually in ventilated chambers in a controlled environment with a 12 h light/dark cycle at room temperature (22°C) and had unlimited access to food (Research Diet Inc., New Brunswick, NJ, USA, Purina Diet no. 5008). At 25 weeks, the ZSF1 animals underwent echocardiographic evaluation under anaesthesia (sevoflurane 8% and 1–2.5% for induction and maintenance, respectively) to evaluate hypertrophy as well as diastolic and systolic function as previously described (Hamdani *et al*. 2013). Echocardiographic assessment (Table 2) demonstrated normal systolic function in ZSF1 lean and obese rats. The obese group displayed more hypertrophy than their lean counterparts, which progressed to diastolic left ventricular (LV) dysfunction, evident from a restrictive LV inflow signal, higher E/E′, increased left atrial area and impaired relaxation (prolonged isovolumic relaxation time).

### Preparation of polyacrylamide gels

**Figure 1:**
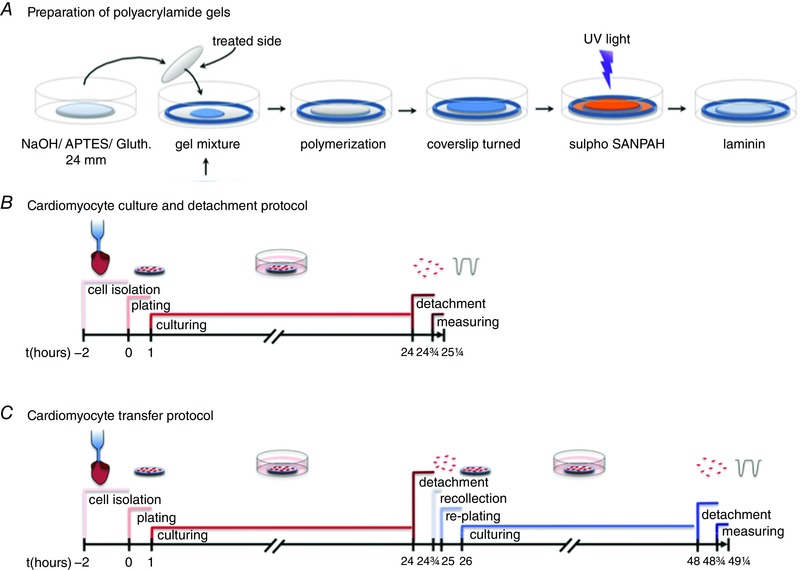
Preparation of gels of tuneable stiffness in a culture dish and experimental timeline *A*, 24 mm coverslips were treated with NaOH, APTES and Gluth (glutaraldehyde). Polyacrylamide gels were polymerized between a glue‐rimmed 30 mm coverslip and the treated side of the 24 mm coverslip. After polymerization the 24 mm coverslip, to which the gel was now attached, was turned. Subsequently, the gel was treated with the cross‐linker Sulpho SANPAH under UV light and coated with laminin. *B*, timeline of the cardiomyocyte culturing and detachment‐protocol. *C*, timeline of the cardiomyocyte transfer protocol.

All steps necessary for gel preparation were performed under sterile conditions in a culture hood. First a thin rim was created on the edge of 30 mm round coverslips using non‐toxic liquid‐resistant glue. Next, 24 mm round coverslips were placed in 6‐well plates and treated with 1 m NaOH (30 min), 97% 3‐aminopropyltrimethoxysilane (APTES) (Sigma‐Aldrich, St Louis, MO, USA) (4 min) and 0.25% glutaraldehyde (30 min), respectively (Fig. 1). In between all treatment steps, the 24 mm coverslips were rinsed with sterile water. In particular the unreacted APTES needs to be washed off thoroughly, as this would otherwise react with the glutaraldehyde and cause orange precipitation on the glass. After removal of the glutaraldehyde, the coverslips were air dried and ready for gel attachment.

Gels of different stiffness were prepared by mixing 40% acrylamide solution (Bio‐Rad, Hercules, CA, USA), 2% *N*,*N*′‐methylene‐bis‐acrylamide (Bis) (Bio‐Rad) and distilled water (Table 1). The components were mixed well, but gently to avoid air bubbles in the mixture. Subsequently, 7.5 μl 10% ammonium persulfate and 1 μl tetramethylethylenediamine (TEMED) (Bio‐Rad) were added to the gel mixture.

**Table 1:** Combinations of acrylamide and Bis for creating gels of a specific stiffness

Stiffness (kPa)	40% acrylamide (μl)	2% Bis (μl)	H_2_O (μl)
8	375	20	1105
15	375	50	1075
50	375	90	1035
100	400	400	700

**Table 2:** Echocardiographic characterization of obese and lean ZSF1 rats

	ZSF1 lean	ZSF1 obese	*P* value
LV mass_i_ (g cm^−2^)	0.64 ± 0.04	1.17 ± 0.06	<0.001
Heart rate (beats min^−1^)	320.0 ± 6.7	288.5± 8.4	0.019
Cardiac output (ml min^−1^)	102.2 ± 3.8	147.8 ± 10.3	0.002
Ejection fraction (%)	76.7 ± 5.0	73.6 ± 5.4	NS
EDV_i_ (μl cm^−2^)	1.12 ± 0.13	1.73 ± 0.14	<0.001
ESV_i_ (μl cm^−2^)	0.29 ± 0.05	0.58 ± 0.05	0.002
E/A	1.55 ± 0.08	1.15 ± 0.08	0.008
E/E′	11.68 ± 0.64	14.76 ± 0.53	0.006
Isovolumic relaxation time (ms)	26.2 ± 1.6	31.0 ± 0.8	0.020
Left atrium area (mm^2^)	2.6 ± 0.1	3.6 ± 0.1	<0.001

A linear 15 MHz probe (Sequoia 15L8W) was used to assess left ventricular (LV) mass, indexed to body surface area (i), heart rate, cardiac output, ejection fraction, end‐diastolic volume (EDV), end‐systolic volume (ESV), the ratio between peak early (E) and late (A) waves of pulsed‐wave Doppler mitral flow velocity (E/A), the ratio between peak E wave velocity of pulsed‐wave Doppler mitral flow and peak E′ wave velocity of tissue Doppler at the lateral mitral annulus (E/E′), isovolumic relaxation time and left atrial area, while the ECG was monitored and the rat temperature kept at 38°C.

Immediately after addition of the TEMED, 43 μl of gel mixture was pipetted onto the glue‐edged 30 mm coverslips that had been placed in sterile 6‐well plates (Fig. 1). The glue edge on the 30 mm coverslip prevents any gel solution from running off the coverslip and ensures that all of the solution is enclosed between the two coverslips. A 24 mm coverslip was swiftly placed on top of the gel solution with the NaOH‐APTES‐glutaraldehyde‐treated side facing the gel solution. The gel polymerizes in approximately 45 min after which 2 ml Hepes (0.1 m, pH 8.5) was put on the coverslips. Subsequently, the 24 mm coverslip, to which the gel was now attached, was carefully removed from the 30 mm coverslip using sharp‐tipped forceps and placed back on the 30 mm coverslip with the gel facing upwards (Fig. 1). Subsequently, the gels were kept wet with 0.1 m Hepes solution at all times to prevent drying and stiffening of the gel.

The cross‐linker sulfosuccinimidyl 6‐(4′‐azido‐2′‐nitrophenylamino)hexanoate (Sulpho SANPAH) (Thermo Fisher Scientific, Waltham, MA, USA) was used to allow coating of the gel with laminin. After washing the gels twice with 0.1 m Hepes (pH 8.5), 500 μl Sulpho SANPAH (2.2 μg ml^−1^ in 0.1 m Hepes, pH 8.5) was put on the gel and the Sulpho SANPAH was photo‐activated by UV light exposure for 5 min in which it changed colour from orange to brown (Fig. 1). Subsequently, the gels were rinsed with 0.1 m Hepes and coated with 350 μl laminin (10 μg ml^−1^) (Sigma‐Aldrich). The dishes were incubated overnight at 4°C or in the incubator for 2 h before plating the cardiomyocytes. In all previous steps the glue‐edged 30 mm coverslip remained as a base under the 24 mm gel coverslip to prevent the solutions from running off the gel (Fig. 1). Additionally, 30 mm coverslips were placed in a 6‐well plate and directly coated with laminin to compare experiments in cells cultured on glass.

### Determining gel stiffness

Stiffness of fully hydrated gels was determined using a Piuma Nanoindenter (Optics 11, Amsterdam, the Netherlands) in combination with an indentation probe with a stiffness of 1 N m^−1^ and a tip radius of 44 μm (Optics 11). The Young's modulus was determined for gels of each stiffness. Nine individual measurements were performed on all gels to determine the average gel stiffness.

### Adult rat cardiomyocyte isolation and culturing

Two different digestive enzymes, Liberase TM (Kaestner *et al*. 2009) and Collagenase type II (Sequeira *et al*. 2015), were tested for isolation of adult rat cardiomyocytes. Briefly, adult wild‐type male Wistar rats (200–250 g) and male ZDF1 rats (28 weeks old) were sedated and kept under anaesthesia through isoflurane inhalation. The hearts were quickly removed, cannulated via the aorta and perfused in a Langendorff setup using perfusion buffer. Thereafter, the hearts were perfused with enzyme solution until the tissue was digested sufficiently, the left ventricle was cut into small pieces and triturated with a plastic Pasteur pipette for 3 min in stopping buffer. Subsequently, the cell suspension was filtered through a 300 μm nylon mesh filter and resuspended in CaCl_2_ buffers of increasing Ca^2+^ concentrations to reach a final concentration of 1 mm Ca^2+^.

Isolated adult cardiomyocytes were suspended in plating medium containing Medium 199 (Lonza, Basel, Switzerland), penicillin/streptomycin (1%) and fetal bovine serum (5%). Subsequently, the laminin solution was removed from the matrices and 0.5 ml plating medium, containing the cardiomyocytes, was carefully pipetted on the matrices and the glass coverslips. Cells from wild‐type rats were plated on polyacrylamide gels of 8, 15, 50 and 100 kPa as well as glass. To assess the effect of matrix stiffening in a disease model, cells from obese ZDF1 rats were plated on 15 kPa and 100 kPa substrates and compared to ZSF1 lean cells under the same conditions. In this step, the ends of the pipette tips were cut to increase the tip diameter and reduce cell damage.

One hour after plating, cells that were not attached to the matrices were removed by replacing the plating medium with culture medium that consisted of Medium 199, penicillin/streptomycin (1%) and ITS supplement (Sigma‐Aldrich; composition: insulin, 10 mg l^−1^; transferrin, 5.5 mg l^−1^; and selenium 5 μg l^−1^). The 30 mm glue‐edged coverslips were removed, leaving the 24 mm coverslip with the matrices and attached cells in the culture dish. Subsequently, the cells were cultured at 37°C in humidified air with 5% CO_2_.

As the contractile movement of cardiomyocytes also influences the interaction between the matrix and cardiomyocytes, we aimed to mimic this interaction via electrical stimulation of the cultured cardiomyocytes using a C‐pace cell culture stimulator (IonOptix, Westwood, MA, USA). Cardiomyocytes were electrically stimulated at a pulse voltage of 8 V, a pulse frequency of 0.2 Hz and a pulse duration of 4 ms. Complete filling of the culture dishes with culture medium and refreshing of the medium every 12–14 h markedly enhanced survival of the cardiomyocytes.

### Detachment of cardiomyocytes from the matrix

To allow measurement of cardiomyocyte function independent of the passive inhibition by the matrix the cells were detached from the hydrogel. We first tried commonly used enzymatic detachment methods like Trypsin (Lonza), Accutase (Sigma‐Aldrich) and Detachin (Genlantis, San Diego, CA, USA), which target the proteins that enable the cells to adhere to the matrix. As this process of cell dissociation disrupts the internal integrity of the cell, we found cell dissociation enzymes are lethal to adult cardiomyocytes. A gentler way to detach cells using Enzyme Free Cell Dissociation Buffer (Gibco, Thermo Fisher Scientific, Waltham, MA, USA) was not potent enough to release cardiomyocytes. Therefore the commonly used techniques of dissociating cells cannot be used for adult cardiomyocytes. We solved this problem by dissolving the laminin matrix rather than targeting the adherent proteins of the cardiomyocytes, using Liberase.

Cultured cardiomyocytes were incubated for 45 min with 750 μl Liberase medium containing 0.3 mg ml^−1^ Liberase TM (Roche, Basel, Switzerland) in culture medium. During the 45 min, ∼80% of the cells detached from the matrix, but careful knocking against the dish could loosen additional cells. To remove the Liberase from the cardiomyocyte suspension, the cells were carefully transferred to a small tube and centrifuged for 2 min at 20 *g*. The medium was removed from the cell pellet and the cardiomyocytes were carefully re‐suspended in culture medium.

### Cardiomyocyte shortening and Ca^2+^ transients

Cell shortening and Ca^2+^ signalling measurements were performed as previously described (Sequeira *et al*. 2015) in cells derived from 11 separate isolations from wild‐type rats and 7 isolations from ZDF1 rats (2 lean, 5 obese). Briefly, the cardiomyocyte suspension was incubated for 5 min with Fura‐2 acetoxymethyl ester (Fura‐2 AM) (0.2 μg ml^−1^) after which single cardiomyocytes were visualized in a video‐based sarcomere length detection system (IonOptix) at 37°C in culture medium. Cardiomyocytes were selected based on the following criteria: rod‐shaped, no spontaneous contractions, stable sarcomere length of at least 1.6 μm and stable contraction. Upon field stimulation (0.5 Hz, 4 ms, 14 V), cell shortening and Ca^2+^ transients were simultaneously assessed.

### Cardiomyocyte isometric force measurements

The force‐generating capacity of wild‐type single membrane‐permeabilized cardiomyocytes cultured on 15 kPa and 100 kPa matrices were assessed as described previously (van Deel *et al*. 2011). In short, cardiomyocytes from six rats (1–3 cells per rat for each stiffness) were permeabilized with Triton X‐100. Isometric force measurements were performed in activation solutions with different calcium concentrations to determine myofilament maximal force (*F*_max_) as well as myofilament passive force (*F*_pas_), maximal rate of force redevelopment (K_tr_) and Ca^2+^ sensitivity (EC_50_) at sarcomere length 2.2 μm.

### Transferring cells from stiff to normal matrices

Isolated cardiomyocytes from five individual wild‐type rats were cultured for 24 h on 15 kPa and 100 kPa matrices and subsequently detached with Liberase as described above. To wash out the Liberase from the suspended cells, the detached cardiomyocytes were collected in a small tube and centrifuged for 2 min at 20 *g* after which the Liberase medium was removed from the cell pellet and the cardiomyocytes were carefully re‐suspended in 2 ml of plating medium. Subsequently the cardiomyocytes were again centrifuged at 20 *g* for 2 min and the medium was removed and replaced by fresh plating medium. After the cells were centrifuged one final time, the medium was removed until just above the bottom of the tube. Cardiomyocytes from four matrices were combined and re‐plated on laminin‐coated matrices. Cells from the 15 kPa gels were transferred to a new 15 kPa gel. Cells from the 100 kPa gels were transferred to either a 15 kPa gel or a fresh 100 kPa gel. Twenty‐four hours after transfer, the cardiomyocytes were again detached and unloaded cell function and Ca^2+^ signalling were determined.

### Immunofluorescence staining of the microtubule cytoskeleton

Cardiomyocytes from three individual wild‐type rats were cultured for 24 h on 15 kPa, 100 kPa and glass substrates. The cells were fixed in 10% paraformaldehyde in cytoskeleton buffer (10 mm Mes, 138 mm KCl, 3 mm MgCl, 2 mm EGTA, pH 6.1) without prior washing. Subsequently, the cells were labelled with a monoclonal antibody against β‐tubulin (Cell Signaling Technology, Inc., Danvers, MA, USA) to visualize microtubules and stained with wheat germ agglutinin (WGA) to determine cell size. A total of 10–22 cells from matrices of each stiffness were measured. Microtubule density was determined as the average intensity of five Z‐stack images from each cell.

### Western blot procedure to determine tubulin network stability

Protein from cells of five wild‐type rats, cultured for 24 h on gels of 15 kPa, 100 kPa and glass, were isolated using Laemmli buffer and Western blotting analysis was performed. In short, equal amounts of protein were separated on SDS‐PAGE gels, transferred onto nitrocellulose membranes, and probed with anti‐acetylated‐α‐tubulin (Sigma‐Aldrich) and anti‐α‐tubulin (Sigma‐Aldrich).

### Data analysis and statistics

The average of all cells from one rat, cultured on one specific stiffness was considered as one data point. Subsequently, the data obtained from the individual rats were averaged. Cell shortening and Ca^2+^ transients were analysed using IonOptix Software (IonOptix, Westwood, MA, USA) and isometric myofilament forces were analysed using the software program Myo (Dept Physiology, VUmc Amsterdam, the Netherlands). Immunofluorescent images were analysed using Slidebook digital microscopy software (3i, Denver, CO, USA), and Western blots were analysed with ImageQuant TL software (GE Healthcare Life Sciences, Chicago, IL, USA). One‐way ANOVA was performed followed by a Tukey post‐test for sarcomere function and Ca^2+^ handling in wild‐type cells and microtubule data. A Student's *t* test was performed to compare force‐generating capacity and to compare functional measurements of ZSF1 rats. Contractile properties and Ca^2+^ handling of cardiomyocytes from ZSF1 rats were analysed by a two‐way ANOVA test followed by a Tukey post‐test using Prism version 6.0 (Graphpad Software Inc., La Jolla, CA, USA). A value of *P* ≤ 0.05 was considered statistically significant. Data are presented as mean ± SEM.

## Results

### Matrix stiffness

Different combinations of 40% acrylamide and 2% Bis were used to obtain gels of the desired stiffness. The stiffness of the gels was aimed to represent the healthy heart (∼15 kPa) and diseased heart (50–100 kPa) (Berry *et al*. 2006; Engler *et al*. 2008; Wells & Discher, 2008). Additionally, a more compliant gel (8 kPa) and untreated glass coverslips were included. The mixtures of acrylamide and Bis resulting in a stiffness of 8, 15, 50 and 100 kPa are listed in Table 1.

### Liberase digestion is optimal for adult rat cardiomyocyte isolation and culturing

**Figure 2:**
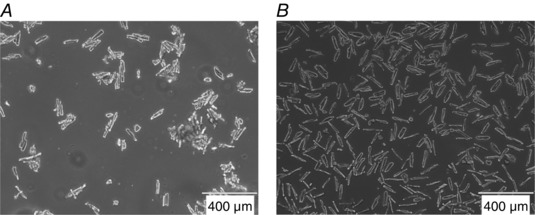
Adult cardiomyocytes in culture after collagenase II or Liberase TM isolation Collagenase digestion (*A*) resulted in a lower yield and survival rate of cells than Liberase TM digestion (*B*). Additionally, collagenase‐isolated cardiomyocytes tend to clump together whereas Liberase digestion resulted in mostly single cells.

We compared two commonly used isolation protocols for adult cardiomyocyte isolation using collagenase and Liberase digestion. Liberase digestion not only gave the highest yield and survival (∼80%) compared to collagenase digestion (∼55%), but also resulted in a higher number of single cells, whereas the collagenase‐treated cells tended to clump together (Fig. 2). When stimulating cardiomyocyte contraction, a cell culture of individual cells is preferred as cells that lie on top of each other will influence each other's contraction pattern. Therefore, we concluded that Liberase digestion is the preferred protocol for isolating adult cardiomyocytes when studying contractile function of cultured cardiomyocytes. We subsequently isolated single cardiomyocytes using Liberase as previously described (Kaestner *et al*. 2009) for functional measurements in cells from 11 wild‐type and 7 ZSF1 rats.

### Matrix stiffening impairs cell shortening and Ca^2+^ signalling in detached intact cardiomyocytes

**Figure 3:**
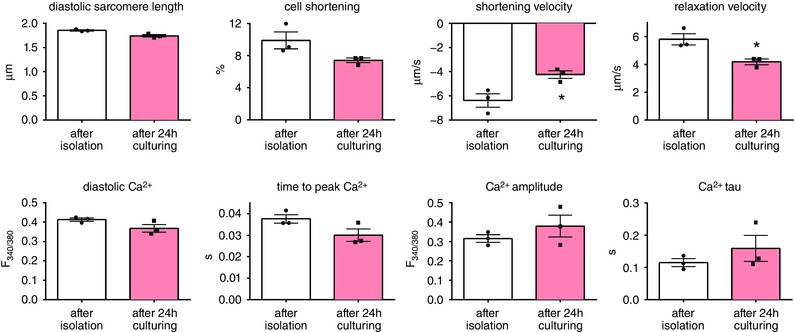
Paired measurements of cardiomyocyte function and Ca^2+^ signalling immediately after isolation and after 24 h of culturing on a 15 kPa gel

Culturing cardiomyocytes for 24 h on matrices of 15 kPa did not significantly affect cell shortening or Ca^2+^ signalling, but contraction velocity and relaxation velocity were both reduced after culturing, compared to cardiomyocyte function measured directly after isolation (Fig. 3). For this reason, we determined the effects of substrate stiffness on cellular performance by comparing all data to measurements obtained in the cells cultured on the healthy 15 kPa gel, isolated from the same rat and cultured under identical conditions.

**Figure 4:**
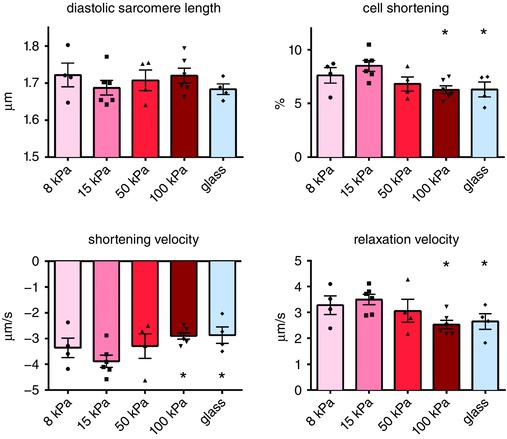
Properties of intact cardiomyocytes cultured on gels of tuneable stiffness Cardiomyocyte cell contraction as well as contraction velocity and relaxation velocity are impaired by matrix stiffening. Cardiomyocytes from 6 rats (15–20 cells per rat for each stiffness) were measured. ^*^ *P* < 0.05 *vs*. 15 kPa.

The ability to detach cultured adult cardiomyocytes from their matrix made it possible to measure cell shortening and Ca^2+^ cycling in intact adult cardiomyocytes independent of passive inhibition by a stiff environment. Cell function and Ca^2+^ handling were measured in detached intact cardiomyocytes that had been cultured on matrices ranging from 8 to 100 kPa and glass. In wild‐type rats, diastolic sarcomere length was unaffected by matrix stiffness while cell shortening as well as velocities of contraction and relaxation were reduced in cells cultured on the stiffer matrices (Fig. 4).

**Figure 5:**
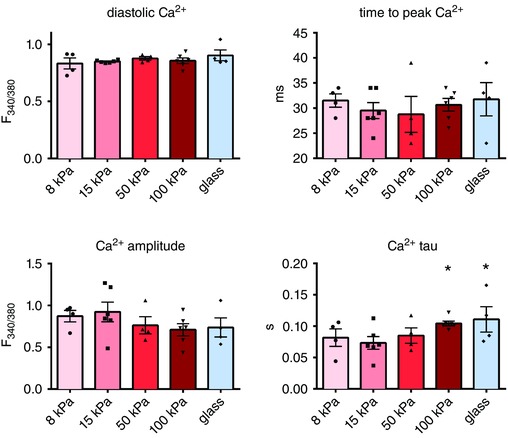
Ca^2+^ signalling of cardiomyocytes cultured on gels of tuneable stiffness Matrix stiffening impairs Ca^2+^ cycling, demonstrated by the increased time constant of diastolic Ca^2+^ (tau). Cardiomyocytes from 6 rats (15–20 cells per rat for each stiffness) were measured. ^*^ *P* < 0.05 *vs*. 15 kPa.

Matrix stiffening did not affect the level of intracellular Ca^2+^. However, in line with the reduction in relaxation speed, the time constant of the diastolic Ca^2+^ transient (tau) was elevated in cardiomyocytes cultured on the stiff matrices, indicating that Ca^2+^ reuptake was slower in these cells (Fig. 5).

### Effects of matrix stiffness on myofilament force‐generating capacity in permeabilized cardiomyocytes

**Figure 6:**
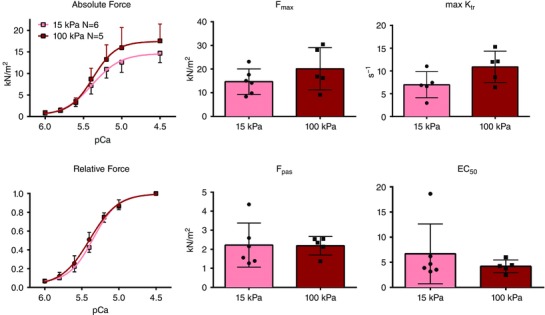
Isometric force development and Ca^2+^ sensitivity in single membrane‐permeabilized cardiomyocytes Maximal force development (*F*_max_), passive force (*F*_pas_) and maximal rate of force redevelopment (K_tr_) were not significantly altered in response to matrix stiffening. The normalized relative force curves further demonstrate that myofilament Ca^2+^ sensitivity (EC_50_) was not affected by matrix stiffening. Cardiomyocytes were isolated from 6 rats and 1–3 cells were measured per stiffness from each rat.

Detachment of cultured cardiomyocytes additionally allowed us to directly measure myofilament force capacity in membrane‐permeabilized cardiomyocytes. Although the maximal generated force (*F*_max_) and maximal rate of force redevelopment (K_tr_) tended to increase in response to matrix stiffening (*P* = 0.24 and 0.09 respectively) this failed to reach statistical significance (Fig. 6). Similarly, we did not observe a significant difference in passive force (*F*_pas_), and the overlap of the 15 kPa and 100 kPa normalized force curves demonstrate that Ca^2+^ sensitivity (EC_50_) was not different between the two groups (Fig. 6).

### Reduction of matrix stiffness reverses stiffness‐induced cardiomyocyte contractile dysfunction

**Figure 7:**
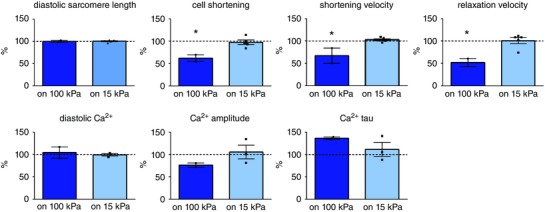
Cardiomyocyte contractile function and Ca^2+^ handling after transfer from a stiff (100 kPa) matrix to another stiff matrix or a ‘healthy’ (15 kPa) matrix Cellular contraction and Ca^2+^ signalling in cardiomyocytes cultured on a 15 kPa matrix were not altered after transfer to another 15 kPa matrix and set to 100% (dotted line). Cardiomyocyte shortening, shortening velocity and relaxation velocity were impaired in cardiomyocytes that were cultured on a 100 kPa matrix and transferred to another 100 kPa matrix, but normalized in cardiomyocytes that were transferred from a 100 kPa to a 15 kPa matrix. Data are presented as percentage change *vs*. corresponding 15 kPa to 15 kPa measurement. Cardiomyocytes from 5 rats (6–21 cells per rat for each stiffness) were measured. ^*^ *P* ≤ 0.05 *vs*. cardiomyocytes transferred from a 15 kPa to another 15 kPa matrix.

To determine if matrix stiffness‐induced contractile alterations are reversible, we transferred cultured cardiomyocytes from a stiff matrix (100 kPa) to a matrix of healthy stiffness (15 kPa). Transfer of cells from a 15 kPa matrix to another 15 kPa matrix did not alter cell contraction or Ca^2+^ signalling (not shown). The stiffer matrix (100 kPa) impaired cell shortening, shortening velocity and relaxation velocity in cardiomyocytes transferred from a 100 kPa matrix to another 100 kPa matrix. However, reduction of stiffness by transferring cardiomyocytes from a 100 kPa (stiff) matrix to a 15 kPa (healthy) matrix normalized contractile function in these cells (Fig. 7). This indicates that normalization of matrix stiffness reverses the stiffness‐induced cardiomyocyte dysfunction.

### Microtubule network density and stability

**Figure 8:**
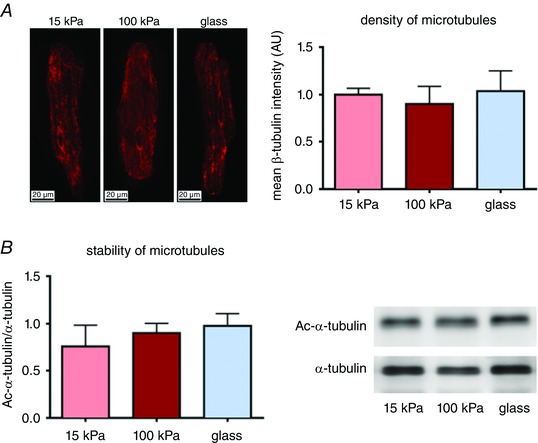
Microtubule density and stability of cardiomyocytes cultured on gels of tuneable stiffness The density and stability of cardiomyocyte microtubules were not affected by stiffening of the substrate. Ten to twenty cells for each stiffness were measured isolated from 3 wild‐type rats for β‐tubulin density determination. Western blot for Ac‐α‐tubulin and α‐tubulin was performed in cells collected from 5 rats. Ac‐α‐tubulin, acetylated α‐tubulin.

In order to evaluate whether changes in the microtubule cytoskeleton contributed to the observed functional adaptations of cardiomyocytes to substrate stiffness, microtubule density was visualized in cardiomyocytes cultured on 15 kPa, 100 kPa and glass substrates. Immunolabelling with anti‐β‐tubulin antibody (red) revealed a fine tubulin network throughout the entire cell and around the nucleus (Fig. 8 *A*). No effect of matrix stiffness on β‐tubulin density was observed. Similarly, microtubule network stability, as determined through α‐tubulin acetylation, was not affected by substrate stiffness (Fig. 8 *B*).

### Effect of matrix stiffness on cardiomyocytes from lean and obese rats

To define the effect of matrix stiffness in a disease model, a comparison was made between cardiomyocytes from ZSF1 lean control and ZSF1 obese HFpEF rats cultured on substrates with a healthy (15 kPa) and a diseased (100 kPa) stiffness.

**Figure 9:**
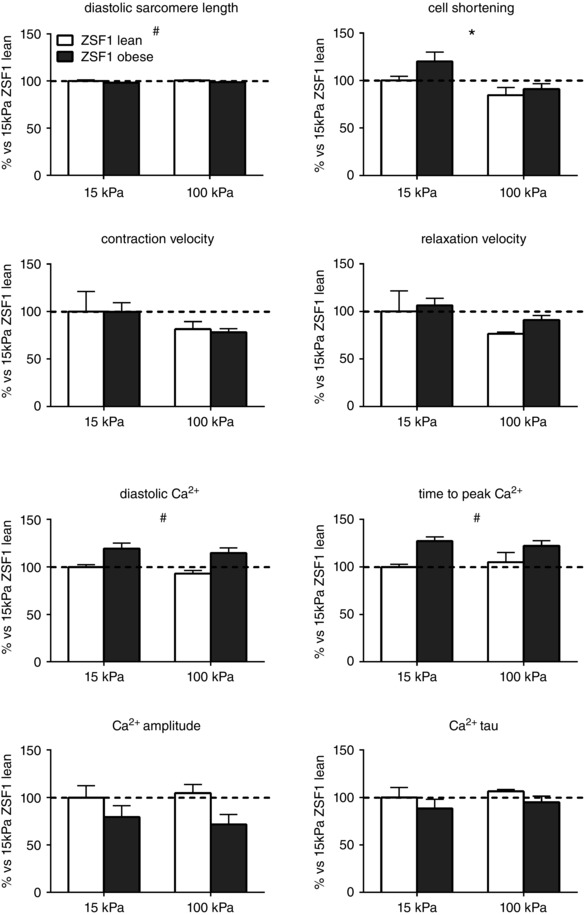
Contractile properties and Ca^2+^ handling of cardiomyocytes from ZSF1 lean control and ZSF1 obese HFpEF rats, cultured on 15 kPa, 100 kPa and glass substrates Enhanced cell shortening of cardiomyocytes from ZSF1 obese rats was associated with disturbed Ca^2+^ handling demonstrated by elevated diastolic Ca^2+^ levels and prolonged time to peak Ca^2+^. ^*^ *P* < 0.05 15 kPa *vs*. 100 kPa, ^#^ *P* < 0.05 ZSF1 lean *vs*. ZSF1 obese.

Figure 9 illustrates that diastolic sarcomere length and Ca^2+^ signalling were not affected by matrix stiffness in cells from lean and obese ZSF1 rats, while substrate stiffening reduced cell shortening, and tended to reduce contraction velocity (*P* = 0.08) and relaxation velocity (*P* = 0.06) in lean and obese rats. Interestingly, the reduced cell shortening in response to matrix stiffening (15 *vs*. 100 kPa) was two times larger in obese ZSF1 cells than in lean cardiomyocytes (29% *vs*. 15%), indicating a direct effect of matrix stiffness on cardiomyocyte behaviour. Additionally, ZSF1 obese cardiomyocytes were characterized by a small but significant reduction in diastolic sarcomere length, higher diastolic Ca^2+^ levels and a prolonged time to peak Ca^2+^ compared to cells from lean rats, which was independent of matrix stiffness.

## Discussion

We created matrices of tuneable stiffness, mimicking the stiffness of the healthy and the diseased heart, to study the interaction between adult cardiomyocytes and the mechanical properties of their surroundings. The ability to detach the cells from the matrices before functional measurements allowed us to measure alterations in cell shortening and Ca^2+^ signalling in cultured intact adult cardiomyocytes independent of passive inhibition by a stiff matrix. Additionally, this novel approach of detaching cultured adult cardiomyocytes enabled us to measure myofilament force characteristics in permeabilized cells, and opened up the possibility to transfer adult cardiomyocytes from one matrix to another. The ability to create any desired stiffness, using a method that does not require specific equipment or skills and is reproducible, makes this a very easy to use and reliable tool for investigating the impact of matrix stiffness on cellular behaviour.

We found that matrix stiffening reduced cardiomyocyte shortening which closely correlated with impaired Ca^2+^ signalling. However, we did not observe a significant effect of matrix stiffness on myofilament force development. This indicates that the observed reduction in cell shortening is probably caused by impaired Ca^2+^ cycling. Additionally, we show that improving matrix characteristics, by decreasing its stiffness, reverses the matrix stiffening‐induced cellular changes.

In hypertrophic cardiomyocytes, an increase in the microtubule network as well as enhanced interaction of microtubules and the sarcomere, contribute to cellular stiffness and impaired cardiomyocyte contraction (Gomez *et al*. 2000; Cooper, 2006; Robison *et al*. 2016). Similarly, enhanced microtubule density could have contributed to the negative effects of matrix stiffness on cardiomyocyte contractility observed in the current study. However, both microtubule density and stability were not significantly affected by matrix stiffness, implying that the matrix stiffness‐induced reduction in cardiomyocyte function was not related to alterations in the microtubule network.

Previously we reported that hypophosphorylation of the myofilament protein titin contributes more to myocardial stiffness than ECM changes in the HFpEF ZSF1 rat model (Hamdani *et al*. 2013). In addition, the elevated diastolic Ca^2+^ levels observed in the present study in ZSF1 obese cardiomyocytes may contribute to the HFpEF phenotype.

The major matrix stiffness‐mediated effect was seen on cardiomyocyte shortening. Interestingly, the stiffening‐mediated effect on cell shortening was larger in HFpEF ZSF1 obese compared to the ZSF1 lean cardiomyocytes. These data indicate that cells from a diseased (HFpEF) model are more sensitive to a change in matrix stiffness compared to healthy (lean) rat cells.

### Therapeutics targeting matrix pathology

Therapeutic approaches aiming to decrease cardiac stiffness could potentially stop or even reverse the progression of pathological remodelling and dysfunction in the diseased heart. Several animal studies revealed that therapeutics targeting ECM proteins not only reduce collagen production or break advanced glycation end‐products (AGEs) bonds (Liu *et al*. 2003), but also halt cardiac remodelling and preserve cardiac function (Piek *et al*. 2016). In patients with hypertensive heart disease, angiotensin‐converting enzyme inhibition regresses myocardial stiffness and fibrosis, and improves cardiac diastolic function (Brilla *et al*. 2000; Diez *et al*. 2002). However, it is difficult to estimate how much of the effect of these pharmaceuticals is related to reducing extracellular stiffness. A clinical trial targeting exclusively fibrosis‐induced stiffness showed that treatment of patients with an antagonist for the receptor of interleukin‐1 (a mediator of fibrosis) did not rescue cardiac function (Turner, 2014). Improved insight into the impact of cardiomyocyte–ECM interaction in cardiac disease is pivotal for the development of drugs that effectively reduce cardiac stiffness.

### Cardiomyocyte function in *in vitro* models for matrix stiffening

The effects of matrix stiffness on cardiomyocyte contraction have been studied previously but with inconsistent results. One study showed that matrix stiffness did not affect cardiomyocyte contraction (Hersch *et al*. 2013), whereas others observed a reduced cell shortening (Bhana *et al*. 2010; Jian *et al*. 2014; McCain *et al*. 2014). One study found reduced cardiomyocyte shortening after 24 h which was improved after 48 h of culturing on a stiff matrix (Galie *et al*. 2013). In these studies, the cells were attached to their matrices during cell measurements. Consequently, cellular changes, like impaired sarcomere function in response to matrix stiffness, are possibly obscured by the passive mechanical properties of the matrices in these models. Our results show that, independent of the passive inhibition of contraction by a rigid matrix, stiffening of the matrix actively induces cellular adaptations that result in ∼25% reduced cardiomyocyte shortening.

In addition to cardiomyocyte contraction some studies determined the impact of matrix stiffness on cardiomyocyte force development using traction force microscopy. An important aspect of this technique is that it attempts to correct for matrix stiffness and thus determines cardiomyocyte function independent of matrix properties. However, although ingenious, traction force microscopy requires multiple calculations and assumptions to correct for differences in surface stiffness and determine generated force. This possibly contributed to the dissimilar results obtained in these studies. Contraction force is described to go down with increasing matrix stiffness (Jacot *et al*. 2008), but also to remain unaltered within the physiological and pathological range (Bajaj *et al*. 2010; Hersch *et al*. 2013). Additionally one study reported a non‐significant increase in generated force (McCain *et al*. 2014), and yet another found contraction force to be increased clearly within the range of pathological cardiac stiffness (Bhana *et al*. 2010). Unlike the studies previously performed, we were able to measure myofilament force development in response to matrix stiffness in detached membrane‐permeabilized single cells.

### Immature *vs*. adult cardiomyocytes

Another potential cause for contrasting results in previous research is the diversity of type of cardiomyocytes used. Most studies investigating the interaction between matrix stiffening and cardiomyocyte function rely on stem cell‐derived, embryonic or neonatal heart cells. Compared to adult cardiomyocytes these cells are less challenging to maintain in culture, and embryonic and neonatal heart cells are relatively easy to isolate compared to adult cardiomyocytes. However, a major disadvantage of these cells is that they are in principal structurally and functionally immature (Keung *et al*. 2014; Bedada *et al*. 2016) and lack the characteristic rod‐shape morphology and uniaxially aligned sarcomeres of adult cardiomyocyte when cultured on a regular culture dish (Bedada *et al*. 2016). However, great progress has been made in developing methods in maturing these cells to closely mimic the adult cardiomyocyte (Yang *et al*. 2014). Interestingly, matrix stiffness is one of the regulating cues in directing cardiomyocyte maturation (Herron *et al*. 2016).

### Limitations of the study

Although our polyacrylamide substrates of tuneable stiffness represent the key aspect of cardiac stiffening, an important limitation of the study is that the cardiomyocytes are cultured in a 2‐D *in vitro* model that differs from the 3‐D environment of a whole heart. Differences between 2‐D‐ and 3‐D‐cultured cardiomyocytes have been predominantly studied in embryonic stem cells and neonatal cardiomyocytes (Pontes Soares *et al*. 2012; Mei *et al*. 2014). Non‐surprisingly, most of the differences described in these studies are related to spontaneous beating, cell flattening and cell spreading, which are typical features of immature cardiomyocytes and therefore not observed in our adult cell‐model. Still, a gel‐based 3‐D model (Jian *et al*. 2014) where the entire cardiomyocyte is surrounded by the extracellular environment more closely mimics physiological and pathological cardiac conditions. Therefore, one could speculate that extracellular stiffness would affect cardiomyocyte function even more in a 3‐D culture than in a 2‐D model.

## Conclusion

The current research demonstrates an easy to use method for studying the influence of matrix properties on cardiomyocyte function. Moreover, this paper describes for the first time a model that provides the opportunity to measure matrix‐induced cardiomyocyte adaptations independent of the passive influence of matrix rigidity. Previously it was not possible to separate these two contraction‐influencing factors in cultured adult cardiomyocytes as these cells could not be detached from their matrices. Our novel approach of detached cultured adult cardiomyocytes makes it possible to study the effects of altered matrix stiffness on cardiomyocyte function of healthy and diseased cardiomyocytes, in the absence of other pathological ECM‐related factors. As such, the method described in this paper adds an important tool for studying the functional consequences of extracellular remodelling in cardiac disease and to assess the therapeutic impact of approaches aiming to protect/prevent myofilaments against the deleterious effects of a stiffened ECM.

## Additional information

### Competing interests

None declared.

### Author contributions

E.D.v.D. devised, designed and performed the project, analysed data and wrote the manuscript; A.N. performed experiments and analysed data; I.F.‐P. and D.F. maintained and characterized the ZSF1 rats and edited the manuscript; E.V. helped in development of part of the experiments; M.G. performed experiments; K.K. performed experiments; J.v.d.V. supervised the project, wrote and revised the manuscript. All authors approved the final version of the manuscript and agree to be accountable for all aspects of the work. All persons designated as authors qualify for authorship, and all those who qualify for authorship are listed. Experiments were performed at the VU University Medical Centre, Amsterdam, the Netherlands and the Universidade do Porto, Portugal.

### Funding

This work was supported by the Institute for Cardiovascular Research of the VU University PostDoc grant 2015. We acknowledge the support from the Netherlands Cardiovascular Research Initiative: an initiative with the support of the Dutch Heart Foundation, CVON‐2011‐11 ARENA and CVON2014‐40 DOSIS.
